# KRAS inhibitors: going noncovalent

**DOI:** 10.1002/1878-0261.13341

**Published:** 2022-11-27

**Authors:** Matthias Drosten, Mariano Barbacid

**Affiliations:** ^1^ Molecular Mechanisms of Cancer Program Centro de Investigación del Cáncer (CIC) and Instituto de Biología Molecular y Celular del Cáncer (IBMCC), CSIC‐USAL Salamanca Spain; ^2^ Molecular Oncology Program Centro Nacional de Investigaciones Oncológicas (CNIO) Madrid Spain

**Keywords:** colorectal cancer, combination therapies, KRAS^G12D^, noncovalent binding, pancreatic ductal adenocarcinoma

## Abstract

*KRAS* ^G12D^ is the most frequent *KRAS* mutation in human cancer with particularly high frequencies in pancreatic and colorectal cancer. Informed by the structure of the KRAS^G12C^ inhibitor adagrasib, Hallin et al. have now, through multiple rounds of structure‐based drug design, identified and validated a potent, selective, and noncovalent KRAS^G12D^ inhibitor, MRTX1133. This study demonstrated that MRTX1133 inhibited both the inactive and active state of KRAS^G12D^ and showed potent antitumor activity in several preclinical models of pancreatic and colorectal cancer, especially when combined with cetuximab, a monoclonal antibody against the EGFR, or BYL‐719, a potent PI3Kα inhibitor.

## Graphical abstract

A new study by Hallin et al. has identified and validated a potent, selective, and noncovalent KRAS^G12D^ inhibitor, MRTX1133. This inhibitor shows potent antitumor activity in several preclinical models of pancreatic and colorectal cancer, especially when combined either with cetuximab, a monoclonal antibody against the EGFR, or with BYL‐719, a potent PI3Kα inhibitor.

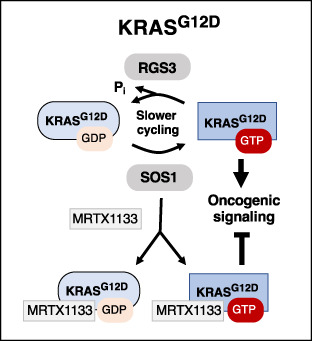

**Abbreviations**
CRC: colorectal carcinoma
GAP: GTPase‐activating protein
GDP: guanosine diphosphate
GMPPCP: β,γ‐Methyleneguanosine 5′‐triphosphate sodium salt
GTP: guanosine triphosphate
IC50: half maximal inhibitory concentration
IP: intraperitoneal
IV: intravenous
ORR: objective response rate
RBD: RAS‐binding domain

## 1 A historical perspective of KRAS‐targeting drugs

*KRAS* oncogenes have been identified in a quarter of all human tumors and appear with high prevalence in some of the most lethal types of cancer such as pancreatic ductal adenocarcinoma (PDAC, 95%), colorectal carcinoma (CRC, 50%), and lung adenocarcinoma (LUAD, 30%). Yet, KRAS oncoproteins were considered undruggable for decades and patients with *KRAS*‐mutant tumors remained excluded from personalized medicine approaches [1]. This notion changed recently thanks to the identification of a previously unrecognized pocket in the switch‐II region (switch‐II pocket) of KRAS. Moreover, by taking advantage of the reactive cysteine residue in one of the mutant KRAS isoforms (KRAS^G12C^), Shokat and colleagues developed the first compound to directly block a KRAS oncoprotein [2]. Since the switch‐II pocket is only accessible when KRAS^G12C^ is bound to GDP and therefore inactive, binding of a covalent inhibitor requires a substantial degree of nucleotide cycling to effectively block this oncoprotein. Indeed, KRAS^G12C^ retains a significant level of nucleotide cycling despite its insensitivity to classical GTPase‐activating protein (GAP)‐stimulated GTP hydrolysis which in this case is mediated via the noncanonical GAP RGS3 [3].

**Fig. 1:**
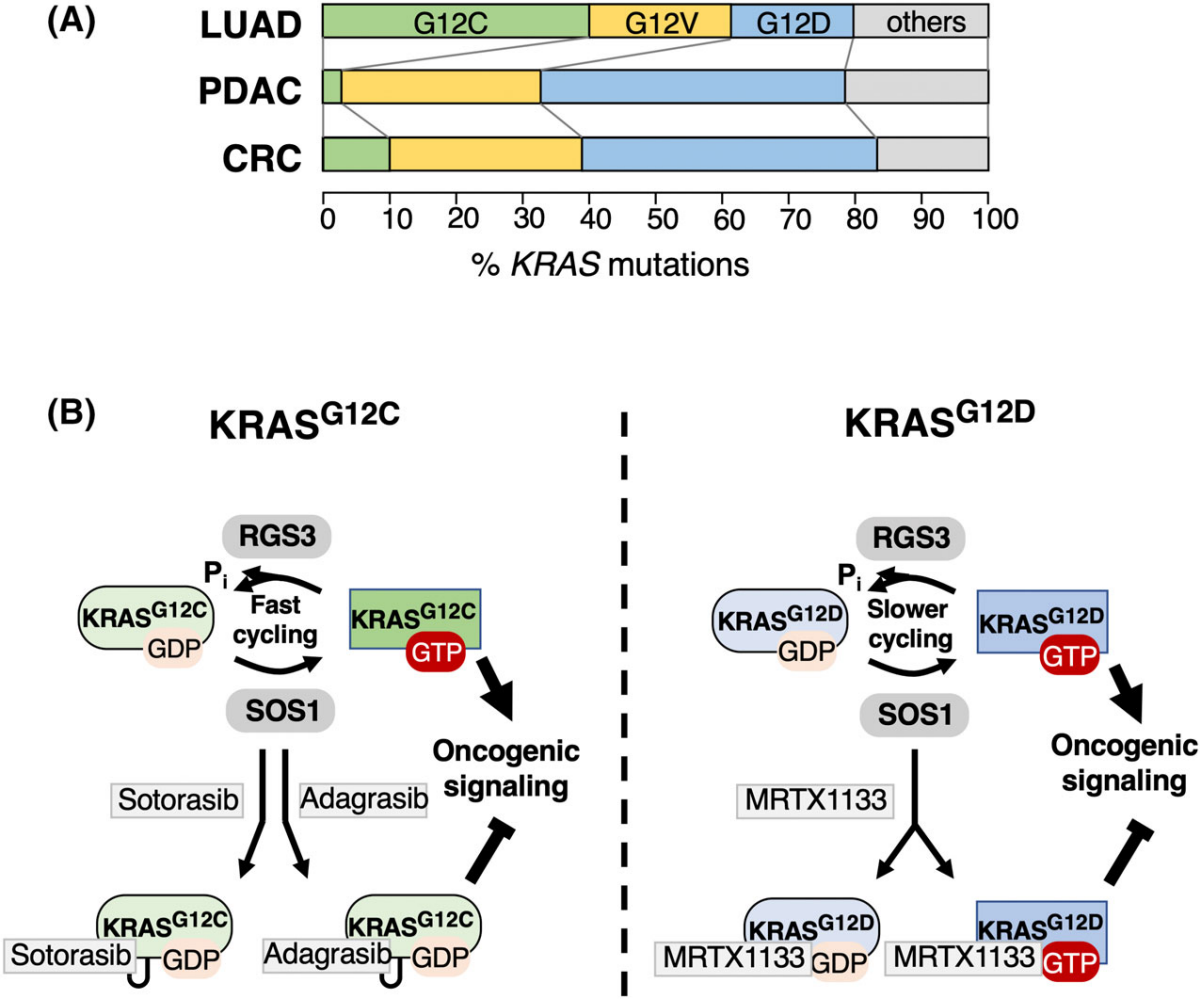
MRTX1133 inhibits KRAS^G12D^. (A) Frequency of mutant *KRAS* alleles in lung adenocarcinoma (LUAD), pancreatic ductal adenocarcinoma (PDAC), or colorectal carcinoma (CRC). G12C mutations are indicated in green, G12V mutations in yellow and G12D alleles in blue. All other mutations are represented in gray. (B) Left: KRAS^G12C^ inhibition by sotorasib and adagrasib. KRAS^G12C^ is characterized by fast nucleotide cycling where GTP hydrolysis is catalyzed by the noncanonical GAP RGS3. Sotorasib and adagrasib bind to the switch II pocket in the inactive, GDP‐bound state of KRAS^G12C^ and form covalent interactions with the reactive cysteine residue. Right: KRAS^G12D^ inhibition by MRTX1133. KRAS^G12D^ has two‐ to three‐fold slower cycling rates than KRAS^G12C^. MRTX1133 binds both GDP‐ and GTP‐bound KRAS^G12D^ with high affinity without the requirement for covalent interactions.

Further efforts in drug development in the following years led to the development and subsequent approval of sotorasib (AMG 510), a drug developed by Amgen (Thousand Oaks, CA, USA). Sotorasib forms a covalent bond with the KRAS^G12C^ oncoprotein blocking it in its inactive state and has demonstrated clinical efficacy for a subset of patients with *KRAS* ^G12C^‐mutant LUAD as well as CRC [4]. A second covalent KRAS^G12C^ inhibitor developed by Mirati Therapeutics, adagrasib (MRTX849), recently received the Breakthrough Therapy designation by the FDA [5]. Although the *KRAS* ^G12C^ mutation is particularly frequent in LUAD (40% of *KRAS* mutations, Fig. 1A), its overall prevalence only encompasses 13% of all *KRAS*‐mutant tumors, indicating that there is an urgent requirement to target other mutant isoforms.

The most prevalent *KRAS* mutation in human cancer is *KRAS* ^G12D^, present in 33% of all cases and the most frequent mutant *KRAS* allele in PDAC (46%, Fig. 1A). Consequently, the development of inhibitors targeting KRAS^G12D^ has always been of particular interest since the identification of the switch‐II pocket. Yet, this mutant isoform lacks the reactive cysteine residue present in KRAS^G12C^, thus imposing a significant challenge to design selective compounds that bind to this mutant isoform in a stable manner. In addition, the GTP hydrolysis rate of KRAS^G12D^ is two to three times lower than that of KRAS^G12C^ [6]. Nevertheless, informed by the structure of the KRAS^G12C^ inhibitor adagrasib, scientists at Mirati Therapeutics were recently able to synthesize, through multiple rounds of structure‐based drug design, a selective, noncovalent KRAS^G12D^ inhibitor (MRTX1133) that is active at concentrations in the low nm range [7]. This drug binds to the switch‐II pocket with extraordinary high affinity, thereby obviating the requirement for covalent interactions (Fig. 1B).

## 2 Validation of the KRAS^G12D^ inhibitor MRTX1133

A more recent study has now evaluated the mechanism of action and antitumor activity of MRTX1133 [8]. First, the authors performed a series of assays to validate the binding efficacy of the drug to KRAS^G12D^ when compared with wild‐type KRAS. Homogenous time resolved fluorescence (HTRF) as well as surface plasmon resonance (SPR) assays confirmed an IC50 of < 2 nm and a binding KD of 0.2 pm, which are approximately 700‐fold more selective for KRAS^G12D^ over wild‐type KRAS. In addition, MRTX1133 was able to prevent binding of a RAF1 RBD peptide to KRAS^G12D^ preloaded with the nonhydrolyzable GTP analog GMPPCP with an IC50 of 9 nm. Together with the resolved structure of MRTX1133 associated with KRAS^G12D^ bound to GDP or to GMPPCP, the authors observed a conformational change in the switch I and II regions that was incompatible with effector binding. Thus, this indicated that MRTX1133 inhibited both inactive and active KRAS^G12D^ states (Fig. 1B). Although this compound inhibited the inactive form with higher potency, its additional activity against the active form is likely to contribute to its higher overall potency.

The authors also tested the cellular activity of MRTX1133 in cell lines carrying the G12D mutation in *KRAS* [8]. MRTX1133 inhibited downstream signaling pathways in a concentration‐dependent manner with an IC50 of < 3 nm. Moreover, this inhibitory effect was maintained for up to 48–72 h, at least when applied at higher concentrations. The authors also found that MRTX1133 inhibited ERK phosphorylation and cell growth in 2D as well as 3D cultures in 24 out of 25 *KRAS* ^G12D^‐mutant cell lines tested. In contrast, most non‐*KRAS* ^G12D^‐mutant cell lines were not inhibited at all and only a few of them responded at higher concentrations. More importantly, MRTX1133 was active in mouse xenograft tumors in the range of 10–30 mg·kg^−1^. Of note, when tested in a series of 25 human cell line‐ and patient‐derived xenografts (PDX) at 30 mg·kg^−1^, 11 of them displayed tumor regression rates of > 30%. Interestingly, although 73% of PDAC models responded to the treatment, only 25% of CRC models were affected. Whether this holds true in the clinic remains to be determined, but it could be a consequence of the fact that mutant *KRAS* acts as a primary driver in PDAC but not in CRC [8]. Finally, MRTX1133 showed a poor bioavailability when applied orally. Nevertheless, it was effective when administered via IP or IV routes. Whether this limitation will affect the clinical utility of this compound remains to be determined. Yet, data recently presented at the NCI RAS Initiative Symposium held in Frederick, MD suggested that formulation strategies to enhance oral absorption and/or increase IV half‐life may increase the probability of augmenting the efficacy in the clinic.

Based on the limited efficacy in some of the preclinical *in vivo* models, the authors set out to explore factors that constrain the response to MRTX1133 as well as to identify collateral dependencies that could maximize its efficacy. To this end, they conducted a CRISPR/Cas9 sgRNA library screen both *in vitro* and *in vivo*. These experiments revealed several tumor suppressor genes such as *PTEN*, *KEAP1*, *NF1*, or *RB1* that conferred at least partial resistance to MRTX1133. Interestingly, *KEAP1* also scored strongly in one of the *in vivo* models of PDAC, suggesting that *KEAP1* could be a key modifier of antitumor response.

Since KRAS inhibition as a monotherapy does not result in prolonged tumor regression in lung cancer [4, 5], the authors anticipated that combination therapies would likely increase the therapeutic benefit of MRTX1133. The results of the CRISPR/Cas9 sgRNA library screen suggested that, among others, inhibition of EGFR and PTPN11 (SHP2) could synergize with MRTX1133. Informed by these results, the authors selected several compounds that could target these and other proteins and tested whether they could observe a synergistic effect with MRTX1133. Interestingly, combinatorial treatment with MEK, ERK, SHP2, or SOS1 inhibitors did not substantially enhance the activity of MRTX1133 to the same extent previously observed with KRAS^G12C^ inhibitors. However, the HER2 family inhibitors, afatinib and cetuximab, as well as the selective PI3Kα inhibitor, BYL‐719, did show a synergistic effect in PDAC and CRC cell lines. Based on these results, the authors combined MRTX1133 with cetuximab, an EGFR inhibitor approved for *KRAS* WT CRC, and with the PI3Kα inhibitor BYL‐719. These combinations were even efficient in models in which either drug as a monotherapy had no effect. Importantly, both drug combinations did not cause significant body weight loss in mice when used at effective doses, suggesting that there could be therapeutic windows when used in human patients.

The findings described by Hallin et al. may have a significant impact for patients with *KRAS* ^G12D^‐mutant tumors [8]. Until now, the therapeutic options for patients with *KRAS* ^G12D^‐positive PDAC were limited to old chemotherapy strategies, such as gemcitabine, 5‐fluorouracil, or taxanes. PDAC is one of the types of cancer with the lowest survival rates and accumulating evidence indicates that mutations in *KRAS* are an early event that drives this disease. Hence, a drug that could block the initiating event in those PDAC patients with *KRAS* ^G12D^ mutations could lead to substantially improved patient survival. As a proof‐of‐principle, recent clinical data with sotorasib (21% ORR) and adagrasib (50% ORR) in *KRAS* ^G12C^‐mutant PDAC provide initial evidence, although *KRAS* ^G12C^ mutations comprise less than 2% of *KRAS*‐mutated PDAC (Fig. 1A) [9, 10].

Several mechanisms have been described that, at least in cell culture and mouse models of PDAC, allow survival of tumor cells in the absence of mutant *KRAS* [11, 12]. This is not unexpected since lung cancers treated with KRAS^G12C^ inhibitors are also capable of rapidly developing resistance. Therefore, combination therapies are likely to be more effective and the current study by Hallin et al. already proposes several potent combinations [8]. In contrast to PDAC, *KRAS* mutations are usually not considered an initial driving event in CRC, instead being responsible for the progression of adenomas to malignant carcinomas. This property might be one of the reasons for the limited effect of KRAS^G12C^ inhibitors in CRC patients [13]. Whether the combinatorial therapies of MRTX1133 outlined in this study will increase the efficacy of this novel KRAS inhibitor remains to be determined.

Finally, it will be of great interest to see how MRTX1133 will perform in clinical trials. Based on prior experience with sotorasib or adagrasib, it is likely that combination therapies will be much more effective. Until then, additional preclinical studies will shed light on potential mechanisms of resistance and guide clinicians to select appropriate combination therapies. Interrogation of resistance mechanisms in patients treated with sotorasib or adagrasib revealed secondary mutations in *KRAS* that prevented these drugs from binding to the switch‐II pocket, as well as mutations in effectors of related signaling pathways such as *EGFR*, *BRAF* or activating mutations in other *RAS* paralogs [14, 15]. Given the high affinity binding mode of MRTX1133, similar mutations, either preventing high‐affinity binding to the switch‐II pocket or activating other signaling pathways, can be expected. Yet, most tumors acquire resistance without the presence of novel mutations [14, 15]. Understanding the molecular mechanisms responsible for the appearance of resistance will provide new treatment avenues to increase the clinical efficacy of these compounds. Regardless of these potential limitations, the development of MRTX1133 is undoubtedly a major step forward toward the implementation of much‐needed effective therapies for patients with *KRAS* ^G12D^‐mutant tumors.

## Conflict of interest

The authors declare no conflict of interest.
